# Pharmacokinetic and pharmacodynamic comparison of two "pegylated" interferon alpha-2 formulations in healthy male volunteers: a randomized, crossover, double-blind study

**DOI:** 10.1186/1471-2210-10-15

**Published:** 2010-11-23

**Authors:** Idrian García-García, Carlos A González-Delgado, Carmen M Valenzuela-Silva, Alina Díaz-Machado, Marisol Cruz-Díaz, Hugo Nodarse-Cuní, Orlando Pérez-Pérez, Cimara H Bermúdez-Badell, Joel Ferrero-Bibilonia, Rolando Páez-Meireles, Iraldo Bello-Rivero, Fidel R Castro-Odio, Pedro A López-Saura

**Affiliations:** 1 Clinical Trials Division, Center for Biological Research, Havana, Cuba; 2 National Center for Toxicology, "Carlos J. Finlay" University Hospital, Havana, Cuba; 3 Quality Control Direction, Center for Genetic Engineering and Biotechnology, Havana, Cuba; 4 Development Direction, Center for Genetic Engineering and Biotechnology, Havana, Cuba

## Abstract

**Background:**

Interferon (IFN) alpha conjugation to polyethylene glycol (PEG) results in a better pharmacokinetic profile and efficacy. The aim of this study was to compare the pharmacokinetic, pharmacodynamic and safety properties of a new, locally developed, 40-kDa PEG-IFN alpha-2b preparation with a reference, commercially available PEG-IFN alpha-2a in healthy male volunteers.

**Methods:**

A randomized, crossover, double-blind study with a 3-weeks washout period, was done. A single 180 micrograms PEG-IFN alpha-2 dose was administered subcutaneously in both groups. Sixteen apparently healthy male subjects were included. Serum PEG-IFN concentration was measured during 336 hours by an enzyme immunoassay (EIA). Other clinical and laboratory variables were used as pharmacodynamic and safety criteria.

**Results:**

The pharmacokinetic comparison by EIA yielded a high similitude between the formulations. In spite of a high subject variability, the parameters' mean were very close (in all cases p > 0.05): AUC: 53623 vs. 44311 pg.h/mL; Cmax: 333 vs. 271 pg/mL; Tmax: 54 vs. 55 h; half-life (t_1/2_): 72.4 vs. 64.8 h; terminal elimination rate (lambda): 0.011 vs. 0.014 h^-1^; mean residence time (MRT): 135 vs. 123 h for reference and study preparations, respectively. There were no significant differences with respect to the pharmacodynamic variables either: serum neopterin and beta-2 microglobulin levels, stimulation of 2'5' oligoadenylate synthetase expression, and serum IFN antiviral activity. A strong Spearman's rank order correlation (p < 0.01) between the pharmacokinetic and pharmacodynamic concentration-time curves was observed. Both products caused similar leukocyte counts diminution and had similar safety profiles. The most frequent adverse reactions were leukopenia, fever, thrombocytopenia, transaminases increase and asthenia, mostly mild.

**Conclusions:**

Both formulations are fully comparable from the pharmacokinetic, pharmacodynamic, and safety profiles. Efficacy trials can be carried out to confirm clinical similarity.

**Trial registration:**

Registro Público Cubano de Ensayos Clínicos RPCEC00000039.

## Background

Binding of interferon (IFN) alpha to polyethylene-glycol (PEG) represented a remarkable step forward to face the major drawbacks of the conventional IFN formulations. The slow clearance of PEG-IFN alpha maintains viral replication inhibitory concentrations longer time, leading to more effectiveness and fewer administrations. On the other hand, the circulating IFN peak levels are much lower, therefore less intense adverse reactions are induced [1,2].

Two separate versions of PEG IFN alpha-2 have been developed, in correspondence to the existent IFN alpha preparations. Despite the almost identity of IFN alpha-2a and alpha-2b molecules (only one aminoacid different in position 23, not related to the receptor binding site), the polyethylene glycol (PEG) used for each one is different, which supposes differences in their clinical use. The product PEG IFN alpha-2a (PEGASYS^®^), contains a 40-kDa branched PEG, while the PEG IFN alpha-2b (PEG-INTRON^®^) contains a linear 12-kDa PEG. The molecular size of the attached PEG molecule correlates with the protein's *in vivo *half-life. Both products have been approved for the treatment of patient with chronic hepatitis C. Monotherapy with PEG-IFN led to 40% sustained virological response. Afterwards, their combination with ribavirin was approved, rising the responders to 61% [3]. Pegylated interferon is also indicated for the treatment of chronic hepatitis B [4].

Recently, a new conjugate of IFN alpha-2b with 40-kDa branched PEG was obtained in order to cope with nationwide needs. This conjugate demonstrated appropriate thermal stability and less susceptibility to degradation by proteases than the unmodified protein. Additionally, a significantly longer half-life was observed in animal models [5]. The objective of this study was to compare its pharmacokinetic, pharmacodynamic and safety profiles with those of a similar molecular weight, commercially available, reference preparation. PEG IFN alpha-2a was chosen because it was expected that the pharmacokinetics of the experimental molecule should be closer to it than to commercially available PEG IFN alpha-2b which has a different pegylation. Classical IFN-inducible biological markers (neopterin, β2-microglobulin, and 2'5' oligoadenylate synthetase) as well as the antiviral activity in serum were used as indicators of their pharmacodynamic action.

## Methods

A randomized, crossover, double-blind study with a three-weeks washout period was carried out at the National Center for Toxicology, Havana, Cuba, which is a reference unit for bioavailability and bioequivalence studies. The trial was in compliance with the Helsinki Declaration. The protocol was approved by the Ethics Committee of the National Center for Toxicology, and by the Cuban Regulatory Authority.

### Subjects

Sixteen healthy, male volunteers, who gave their written, informed consent to participate, were included. Individuals were considered healthy if they had no history of chronic diseases, did not suffer any acute illness in the previous 30 days, had no symptoms or signs at the physical examination and laboratory tests, and were negative to HIV and hepatitis B and C virus infections markers in serum. Subjects were not included if they had received treatment with IFN or any other drug that could alter immune functions in the previous 15 days, had been operated in the previous 6 months or had donated blood in the previous 3 months. They were withdrawn from the trial if they abandoned voluntarily, had severe adverse reactions, or if any exclusion criteria arose.

### PEG IFN alpha-2 formulations

Formulation A consisted in a commercially available preparation (PEGASYS^®^, Hoffmann-La Roche, Switzerland) presented in pre-filled syringes containing 180 μg PEG IFN alpha-2a, 0.025 mg polysorbate 80, 4.0 mg NaCl, 5.0 mg benzyl alcohol, 1.31 mg tri-hydrated sodium acetate, 0.023 mg acetic acid, and water for injection to complete 0.5 ml. Formulation B was in vials containing 180 μg PEG IFN alpha-2b (produced at the Center for Genetic Engineering and Biotechnology, Havana), 0.1 mg polysorbate 80, 4.89 mg NaCl, 1.0 mg di-hydrated sodium EDTA, 12.68 mg Na_2_HPO_4_, 3.43 mg NaH_2_PO_4_.2H_2_O, and water for injection to complete 1 ml.

### Study design

The study was not designed to proof similarity; only to compare the pharmacologic properties of the new experimental molecule with an established one. After overnight fasting, sixteen subjects received subcutaneously, on the upper right arm, 180 μg (dose commonly used for the reference preparation in clinics) of one of the PEG-IFN preparations (A or B), in a double-blind, randomized, crossover design with a three-week washout period between treatments. Subjects were randomized, according to a computer-generated simple random number list, to receive one of two treatment sequences (AB or BA). The study was double blinded. As the presentation of the products differed, blinding was kept by loading formulation B vials in syringes coded with the patients' inclusion numbers, and the nurse that administered the products did not participate in the rest of the trial. During each period, individuals were hospitalized during the first 96 hours after the injection under strict medical supervision. Blood sampling and adverse reactions monitoring continued ambulatorily until 336 hours. Antipyretic medication was given orally at the same time as the PEG-IFN injection and every 4 hours thereafter, up to 12 hours or more if needed, in order to mitigate the expected IFN-dependent flu-like syndrome.

### Clinical and laboratory evaluations

Blood samples for serum PEG-IFN concentration determinations were collected by venipuncture before and 6, 12, 24, 36, 48, 60, 72, 84, 96, 120, 168, 216, 264, and 336 hours after injection. Pharmacodynamics was assessed by neopterin and β2-microglobulin (β_2_M) concentrations in serum before and at 6, 12, 24, 48, 72, 96, 120, 168, and 336 hours and by the induction of 2',5' oligoadenylate synthetase (2',5' OAS) mRNA expression before and at 6, 48, 168 and 336 hours. Serum IFN antiviral activity before injection and at peak blood level was also used as pharmacodynamic evaluation. Other hematological (hemoglobin, hematocrit, platelet, and total and differential leukocyte counts) and biochemical (transaminases, creatinine) determinations were taken as safety variables, every 24 hours during the first week and at 336 hours. Patients were regularly checked for vital signs and symptoms during the whole study.

PEG IFN alpha-2 was quantified in serum with a high sensitivity enzyme immunoassay (EIA) kit (Biotrak, GE Healthcare Bio-Sciences). This EIA was previously validated to determine PEG-IFN, despite being originally designed for non-conjugated IFN alpha-2 (result not shown). Neopterin in serum was determined by a commercial EIA kit (HENNING test, BRAHMS Diagnostica GmbH, Berlin, Germany) as well as serum β_2_M (Quantikine^® ^IVD^®^, R&D System, Inc, Minneapolis). IFN antiviral activity titration in serum was done by inhibition of Mengo virus cytopathic effect on HEp-2 cells [6]. Titers were adjusted with a laboratory reference preparation that was calibrated against the international WHO IFN alpha standard 95/566. Blood chemistry and hematological counts were done according to usual clinical laboratory procedures. For all laboratory determinations, the analysts were blind with regard to the identity of the subject.

Quantification of 2',5' OAS expression was performed using a Real-Time PCR for mRNA. Briefly, 2.5 ml human whole blood were collected and centrifuged to pellet nucleic acids in a PAXgene Blood RNA Tube. The pellet was washed and re-suspended, then was incubated with proteinase K. Cell lysate was applied to a PAXgene RNA spin column and purified on silica membrane, the membrane was treated with DNase I, washed and finally RNA was eluted and heat-denatured. Real-Time PCR for purified total RNA was performed using QuantiTect^® ^SYBR^® ^Green PCR (QIAGEN GmbH, Germany), using appropriate primers and samples according to the manufacturer's protocol. In brief, 1 μl of cDNA was added to 10 μl of 2x PCR master mix, 8 μl of RNase-free water, and 0.5 μl of each primer (20 μM), for a total volume of 20 μl. All PCR experiments were performed by triplicate. The ratios between target cDNA and 18S ribosomal RNA were evaluated and expressed as relative amount of 2',5' OAS mRNA expression. The REST 2005 software (Corbett Research, Mortlake, Australia) was used for this quantification.

### Data analysis

The drug disposition data analysis was performed per individual by a non-compartmental method with a combined linear/log - linear trapezoidal rule approach. The linear trapezoidal rule was used up to peak level and the logarithmic trapezoidal rule thereafter. The first-order rate constant associated with the curve terminal (log linear) portion (λ) and terminal half-life (t_1/2_) were estimated by linear regression of the included terminal data points. Time-to-peak values (Tmax) were determined directly from the experimental data as the time of maximum observed level (Cmax) considering the entire curve. Area under the serum concentration-time curve from 0 to 336 hours (AUC_336_) was calculated using the linear/log linear trapezoidal rule. Mean residence time (MRT) was also calculated using the moments of the drug disposition curve. Parameters that were extrapolated to infinity, such as AUC (area under disposition curve) and AUMC (area under first moment of the disposition curve) were computed based on the last predicted value from the linear regression performed to estimate λ and t_1/2_. Other pharmacokinetic parameters were also calculated, such as the systemic clearance (CL), distribution volume (Vd), and the peak to area ratio value (CAV = Cmax/AUC). For a better characterization of the slower absorption and elimination processes due to the pegylation, the parameters mean absorption time (MAT) and half value duration (HVD) were estimated. Some similar kinetic parameters were estimated for the pharmacodynamic markers, corrected for baseline values, neopterin and β_2_M in order to describe the kinetic behavior of the IFN-induced immunological response: AUEC (area under the effect curve), Rmax (maximum response), T(Rmax) (time to reach maximum response), MET (mean effect time), RAV (average response). The WinNonlin professional software (Version 2.1, Pharsight Inc., 1997, NC, USA) was used for all these purposes.

Statistical analyses were done using SPSS for Windows version 15.0, and STATISTICA for Windows version 6.1. Firstly, to test the homogeneity between the treatment groups, the Mann-Whitney's U or Student's t test (depending on the normality assumption) was applied for continuous control variables and the chi-square or Fisher's exact test for the categorical ones. Pharmacokinetic parameters were tested for normal distribution by the Shapiro-Wilk's test and for variance homogeneity by the Levene's test. To compare formulations paired analysis (Student's t test or Wilcoxon's test) depending on the normality assumption were used. Confidence intervals (90%) for the difference between pharmacokinetic parameters were calculated according to Westlake [7]. The Wilcoxon method, proposed by Steinijans [8] was used for the parameters that did not fulfill the normality assumption. The 90% confidence intervals were estimated by the non parametric method proposed by the same authors [8]. The pharmacodynamic variables neopterin and β_2_M were treated similarly. Spearman rank correlation analyses between these AUEC and PEG IFN concentration AUC was done. IFN antiviral activity, 2',5' OAS, vital signs and clinical laboratory variables were treated using paired analysis (Student's t test or Wilcoxon's test) depending on the normality assumption, taking into account Bonferrony's adjustment for multiple comparisons. The calculated significance level (α) is indicated below the corresponding Table. Adverse reactions between formulations were compared using the McNemar's test.

## Results

Sixteen healthy male volunteers were recruited. The demographic and baseline characteristics of the subjects, grouped by sequence of treatment, are shown in Table 1. The hypothesis of homogeneity between the groups was accepted. All the individuals complied with the treatment and evaluations as previewed. Surprisingly, one subject (No.13) presented very low PEG-IFN levels after the administration during both periods and did not have any pharmacodynamic effect either. Therefore he was excluded from the pharmacokinetic and pharmacodynamic analyses and only taken into account for safety. He developed mild fever and moderate leukopenia.

**Table 1:** Demographic and baseline characteristics of the groups of treatment.

Variables	AB sequence N = 8	BA sequence N = 8	Total N = 16
Age (years)	27 ± 4 (24 - 35)	28 ± 5 (21 - 35)	27 ± 4 (21 - 35)
Weight (Kg)	70 ± 11 (60 - 92)	77 ± 12 (58 - 94)	74 ± 12 (58 - 94)
Height (cm)	175 ± 8 (164 - 185)	178 ± 8 (165 - 192)	176 ± 8 (164 - 192)
Body Mass Index	22.9 ± 2.7 (19.0 - 28.4)	24.2 ± 2.6 (20.9 - 29.3)	23.6 ± 2.6 (19.0 - 29.3)
Body surface (m^2^)	1.84 ± 0.29 (1.58 - 2.42)	2.03 ± 0.32 (1.53 - 2.48)	1.94 ± 0.31 (1.53 - 2.48)
Skin color			
White	6 (75%)	7 (87.5%)	13 (81.3%)
Non-white	2 (25%)	1 (12.5%)	3 (18.7%)

Data are reported as mean ± standard deviation (range) or number of patients (%).

### Pharmacokinetic analysis

**Figure 1:**
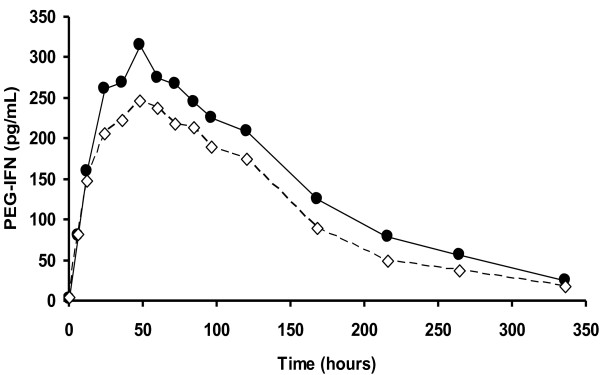
**Average PEG-IFN concentration in serum**. Data correspond to 15 healthy male subjects who received 180 μg of PEGASYS^® ^(solid line) and 40-kDa PEG IFN alpha-2b (dashed line). Standard deviations are not shown for the sake of simplicity of the illustration.

Except for two subjects in the second period with Formulation B, who had 23 and 27 pg/ml, all had low basal serum IFN concentrations (1,8 pg/ml and 2,4 pg/ml before the first and second period, respectively). Table 2 shows the results of the PEG-IFN pharmacokinetic comparisons. The mean values for the parameters were very similar between the formulations despite the subject -dependant variability observed (see SD). A formulation effect was not detected for any of the parameters (p > 0.05 in all cases). All the 90% confidence intervals included the value 1. The average concentration profiles obtained for both formulations were very similar (Figure 1). At 336 hours after the injection, serum PEG-IFN concentrations had returned near to the initial values, so the AUC_336 _obtained covered, in most of the cases, more than 95% of the AUC extrapolated to infinite. No residual effect from the first treatment period on the second period IFN concentration values was found.

**Table 2:** Pharmacokinetic parameters calculated from the PEG-IFN concentration in serum.

Parameter	Formulation A N = 15	Formulation B N = 15	p	90% CI
AUC (pg.h/mL)	53623 ± 37517	44311 ± 26035	0.125	(0.66 - 1.03)
Cmax (pg/mL)	333 ± 205	271 ± 206	0.26	(0.62 - 1.06)
Tmax (h)	48 ± 24	48 ± 48	0.80	(0.78 - 1.33)
λ (h^-1^)	0.011 ± 0.004	0.014 ± 0.008	0.38	(0.90 - 1.52)
t_1/2 _(h)	72.4 ± 28.1	64.8 ± 29.6	0.45	(0.71 - 1.12)
MRT (h)	135 ± 43	123 ± 41	0.38	(0.78 - 1.06)
CAV	0.008 ± 0.003	0.008 ± 0.003	0.56	(0.93 - 1.17)
CL/F (L/h)*	7.9 ± 12.4	7.4 ± 15.6	0.16	(0.57 - 1.70)
Vd/F (L)*	720 ± 1205	841 ± 2130	0.69	(0.61 - 2.23)
MAT (h)	33.0 ± 13.2	29.1 ± 14.0	0.26	(0.77 - 1.15)
HVD (h)	126 ± 66.5	153 ± 113	0.34	(0.89 - 1.56)

Data are reported as mean ± standard deviation, except for Tmax expressed as median ± quartile range.

CI: confidence intervals of the mean Test/Reference ratio

One volunteer (#13) was excluded from the pharmacokinetic comparison (see text)

* Parameters corrected by corporal surface

p: Paired t test between groups, except for Tmax (Wilcoxon's test)

### Pharmacodynamic analysis

**Figure 2:**
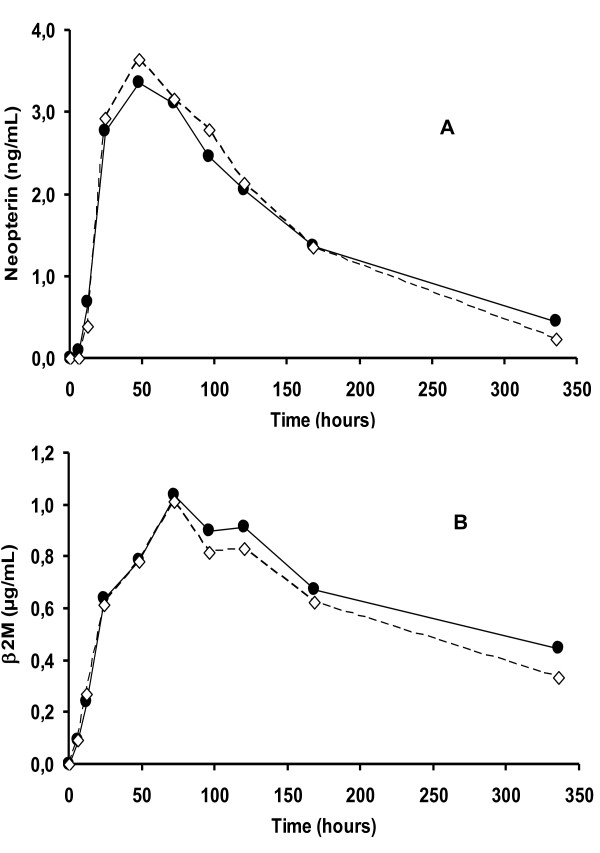
**Increments of the pharmacodynamic markers neopterin and β_2_M**. Data correspond to 15 healthy male subjects who received 180 μg of PEGASYS^® ^(solid line) and formulation of 40-kDa PEG IFN alpha-2b (dashed line) at time 0. (A): Average neopterin concentration in serum, measured by EIA. (B): Average β2-microglobulin concentration in serum, measured by EIA. Standard deviations are not shown for the sake of simplicity of the illustration.

Average serum neopterin increments were very similar for both PEG IFN alpha-2 formulations, with the same profiles (Figure 2A). The induced increments approximately tripled the basal values 48 hours after injection, and then slowly returned to baseline. Figure 2B shows the same high similitude between the formulations with respect to mean serum β2-microglobulin increments, which peaked around 60% from baseline at 72 hours for both formulations. A pharmacokinetic-like analytical procedure was carried out with both variables (Table 3). No difference between the formulations was detected for the estimated parameters, although a high intra-subject variability was again evidenced. The confidence intervals of the test/reference ratios included 1. Both pharmacodynamic markers significantly correlated with the PEG-IFN concentration by EIA for both formulations, according to the calculated AUC and AUEC. A stronger correlation was obtained for β2-microglobulin (Table 4). At the end of the sampling period (336 hours), concentrations of the induced markers (especially β2-microglobulin) had not fully returned to baseline, but this did not determine a residual effect for the second period.

**Table 3:** Descriptive analysis of the kinetics of serum Neopterina and serum β2-microglobulin increments.

Variable	Formulation A N = 15	Formulation B N = 15	p	90% CI
**Neopterin**				
AUEC (ng.h/mL)	500 ± 332	474 ± 257	0.75	(0.71 - 1.26)
Rmax (ng/mL)	3.6 ± 1.9	3.8 ± 2.1	0.79	(0.75 - 1.48)
T(Rmax) (h)	48 ± 24	48 ± 24	0.19	(0.61 - 1.00)
MET (h)	104 ± 36.5	108 ± 34.2	0.66	(0.88 - 1.24)
RAV	0.010 ± 0.007	0.009 ± 0.003	0.865	(0.76 - 1.20)
**β2-microglobulin**				
AUEC (μg.h/mL)	211 ± 145	190 ± 95	0.50	(0.70 - 1.16)
Rmax (μg/mL)	1.2 ± 0.5	1.2 ± 0.5	0.99	(0.83 - 1.22)
T(Rmax) (h)	72 ± 72	72 ± 24	0.10	(0.58 - 1.00)
MET (h)	131 ± 43	161 ± 63	0.09	(1.01 - 1.50)
RAV	0.008 ± 0.005	0.006 ± 0.001	0.37	(0.71 - 1.11)

Data are reported as mean ± standard deviation, except for T(Rmax) expressed as median ± quartile range.

CI: confidence intervals of the mean Test/Reference ratio

One volunteer (#13) was excluded from the pharmacodynamic comparison (see text)

p: Paired t test between groups, except for T(Rmax) (Wilcoxon's test)

**Table 4:** Spearman rank order correlation of area under the effect-time curves (AUEC) for neopterin and β2-microglobulin with the PEG IFN concentration AUC.

Induced protein	Formulation	Spearman R value	p
Neopterin	A	0.554	0.03
	B	0.721	0.002

β2-microglobulin	A	0.857	< 0.001
	B	0.839	< 0.001

Since the induction of the enzyme 2',5' OAS was measured through the relative amount of mRNA expression using Real-Time PCR, an important individual variability was observed. Therefore, parameters could not be rigorously calculated from the experimental data and the analysis was essentially qualitative. 2',5' OAS mRNA levels two or more times with regard to their initial values increased in 14/15 subjects with the Formulation A and 13/15 with the Formulation B. The higher increments were obtained within the first 48 hours. No residual effect was observed.

On the other hand, PEG IFN alpha-2 exhibited a direct antiviral activity. Baseline titers were negligible and most of the individuals increased notably their median serum titers at Tmax by EIA similarly for both formulations (Table 5). Titers correlated significantly with the EIA measured concentration.

**Table 5:** Induction of antiviral activity in serum.

Time	Formulation A N = 15	Formulation B N = 15	Wilcoxon's paired test between formulations
Baseline activity in serum	0.0 ± 1.0 (0.0; 2.5)	1.0 ± 2.0(0.0; 4.0)	p = 0.187
Activity in serum at Tmax	16.0 ± 32.0(4.0; 73.0)	20.0 ± 20.0(0.0; 59.0)	p = 0.513
Wilcoxon's paired test between times	p = 0.001	p = 0.001	

Antiviral titers are expressed in IU/mL. Data are reported as median ± quartile range (range). One volunteer (#13) was excluded from this comparison

### Safety analysis

Adverse events were recorded during the whole study. All the subjects presented at least one event with both formulations (Table 6). There were no differences between formulations concerning the frequency of adverse events, except for ardor at site of injection, which were significantly more frequent with Formulation B. The most frequent events with both formulations were leukopenia (87.5%; due to neutropenia), fever (56.3%), thrombocytopenia (56.3%), increase of transaminases (50%) and asthenia (25%). Most of them were mild, few moderate, being well controlled. Just one subject had a severe leukopenia 24 hours after Formulation B administration in the second period. However, this alteration was preceded by a mild leukopenia during the first period (Formulation A). The subject recovered normal leukocyte count spontaneously, without any other symptom, three weeks after.

**Table 6:** Frequency of adverse events during the study

Adverse event	Formulation A N = 16	Formulation B N = 16	Both formulations N = 16	p (McNemar's test)
Any adverse event	16 (100%)	16 (100%)	16 (100%)	---
Leukopenia*	14 (87.5%)	15 (93.8%)	14 (87.5%)	1.00
Fever	13 (81.3%)	11 (68.8%)	9 (56.3%)	0.69
Thrombocytopenia**	9 (56.3%)	12 (75%)	9 (56.3%)	0.25
Increase of transaminases^!^	10 (62.5%)	11 (68.8%)	8 (50%)	1.00
Asthenia	8 (50%)	6 (37.5%)	4 (25%)	0.69
Headache	6 (37.5%)	7 (43.8%)	2 (12.5%)	1.00
Increase of creatinine^!!^	7 (43.8%)	5 (31.3%)	0	0.77
Ardor at injection site	0	12 (75%)	0	0.0005
Erythema at injection site	5 (31.3%)	3 (18.8%)	2 (12.5%)	0.625
Hypertension	4 (25%)	2 (12.5%)	1 (6.3%)	0.625
Chills	1 (6.3%)	4 (25%)	0	0.375
Myalgias	2 (12.5%)	3 (18.8%)	1 (6.3%)	1.00
Lumbar pain	2 (12.5%)	2 (12.5%)	1 (6.3%)	1.00
Pruritus	3 (18.8%)	1 (6.3%)	1 (6.3%)	0.50
Tachycardia	2 (12.5%)	1 (6.3%)	1 (6.3%)	1.00
Somnolence	1 (6.3%)	1 (6.3%)	0	1.00
Warmth at injection site	2 (12.5%)	0	0	0.50
Hyperesthesia	2 (12.5%)	0	0	0.50
Anorexia	1 (6.3%)	0	0	1.00
Vomiting	0	1 (6.3%)	0	1.00
Arthralgias	0	1 (6.3%)	0	1.00

Data are presented as number of individuals with each adverse reaction (%).

* < 5 × 10^9 ^cells/L; ^** ^< 150 × 10^9 ^cells/L; ^! ^> 100 UI; ^!! ^> 97 μmol/L.

## Discussion

The trial design (sample size, randomization, double blinding, crossover, three-week washout period) complied with international guidelines for pharmacokinetic and pharmacodynamic comparisons between formulations [9-11], which say that a minimum of 12 individuals should be included in this kind of study. The facts that more than 80% of the AUC could be covered by the AUC_336 _obtained and that there was no residual effect on the second period PEG-IFN concentrations indicate that internal validity of the results is high. This is the first pharmacokinetic trial where the sampling period characterization of PEG IFN was extended beyond one week. It is unlikely that the small amounts of baseline endogenous IFN found in few subjects had any influence on the results, since the PEG-IFN concentrations attained after the administration were much higher.

The selected dose, similar to other authors, was adequate, since PEG-IFN levels in serum were easily detected and the treatment was quite well tolerated. The crossover design is usually used in this kind of study with other drugs. It has more statistical power to detect differences. This study is the first comparison of different PEG-IFN's in a crossover, randomized fashion. The 3-weeks washout period between both treatments seemed to be enough regarding the PEG-IFN concentration in blood. However, there was some carryover effect for some hematological variables. Sample size was within the range recommended for this kind of study.

The new 40 Kd PEG IFN alpha-2b fulfilled its aims: slow absorption and elimination rates and PEG-IFN alpha blood levels detectable until 2 weeks after a 180 μg single subcutaneous dose, very similar to the reference product used. In a similar type of study with the native IFN alpha-2b the mean values for t_1/2 _and MRT were 5.9 h and 10.9 h, respectively [12]. After "pegylation" these parameters were 64.8 h and 123 h, respectively, around 11 fold higher, consistent with previous studies of 40 Kd PEG IFN alpha-2a vs. its conventional IFN counterpart in healthy subjects [13,14]. Median Tmax was lower (48 h) than expected (72 h) for both formulations, although PEG-IFN concentrations remained close to peak levels between 36 and 84 hours (plateau). The values found for the parameters MAT (around 30 h) and HVD (around 150 h) corroborate the retardation in the absorption and elimination processes.

Both formulations' pharmacokinetics was highly similar. Differences in means did not exceed 20% for any parameter and dispersions were also similar, although large, which resulted in wide 90% confidence intervals for the ratio between formulations. These differences were due to a high intra- and inter-subject variability, not to a formulation effect. The variability in pharmacokinetic parameters in response to PEG IFN treatment is consistent with previous studies [2,13,15,16]. In some situations, the intra and inter-subject variability can be equally or more important than the variation from an innovator product to a similar. The importance of scaling the established intervals based on the individual comparison and the subject -formulation interaction has been claimed [17].

The pharmacodynamic variables measured in this trial are well-known IFN-induced genes, classical surrogate markers of IFN biological actions. A key mechanism of their antiviral activity is through induction of the enzyme 2'-5' OAS that leads to viral and cellular RNA degradation [18]. Beta2-microglobulin plays an important role in the tumor growth control and metastases [19]. Neopterin is an immune activation mediator of [20]. In this study, both products behaved similarly regarding these pharmacodynamic markers. The increments reached almost 300% for neopterin and approximately 60% for β2 M, 48-72 h after administration, as has been reported with 40 Kd PEG-IFN alpha-2a at the same dose and route of administration [15,16].

The integration of pharmacokinetic and pharmacodynamic characterizations into drug development provides a scientific framework for its rational and efficient application [21,22]. A significant, strong correlation between plasmatic PEG-IFN alpha-2 concentration AUC and the AUEC of the main pharmacodynamic variables was evidenced for both formulations, contrasting with other authors [16]. This correlation is an essential part of the general comparability of pharmaceutical preparations that contain the same or very similar active ingredient [23]. Moreover, given the high complexity of IFN mechanism of action, the relationship between its blood concentration and the biological effects is not so direct. The existing PEG IFN alpha-2a and alpha-2b products have different pharmacokinetic properties, but spread comparable pharmacodynamic profiles [16]. The application of different therapeutic schedules with PEG IFN alpha in hepatitis C patients does not impact on the virological sustained response rate [24]. In a report with 3070 adult patients with chronic hepatitis C (genotype 1), where the classic schedules of these two products, combined with ribavirin, were compared, a similar virological sustained response was obtained [25]. This situation comes from the native IFN alpha, where also similar results have been achieved with very different treatment schemes in several therapeutic indications [26-28].

The IFN titration bioassay is internationally accepted to quantify it. Its main advantage is that it directly measures the agent's main biological activity. However, its precision is low, which can affect the study power to detect differences. Additionally, other cytokines could have antiviral action as well or interfere with the assay. Conjugation of IFN alpha-2 to PEG determines an evident loss of its *in vitro *activity. The antiviral activity of the branched 40-kDa PEG-IFN is only 7% of the linear 12-kDa PEG [29]. This determination was used as another pharmacodynamic indicator but only at baseline and Tmax. Both formulations exhibited similar antiviral activity levels.

One volunteer presented very low PEG-IFN concentrations and had no pharmacodynamic induction with any of the products, being necessarily excluded from the analyses, as an outlier. Interestingly, he presented adverse events such as mild fever and moderate leukopenia. These events may not be mediated by the common type I IFN receptor. White blood cells count reduction immediately after IFN injection can be related to early cortisol release [30] and body temperature rise is related to the interaction of IFN alpha to hypothalamic μ-opioid receptors [31]. This subject is being thoroughly studied.

Concerning safety, flu-like symptoms and hematological count reductions were similar. All the local, systemic and laboratory alterations recorded in this study have been reported under the treatment of PEG IFN alpha-2 [32,33].Except for one episode of leukopenia, the adverse events were mild or moderate. All of them disappeared easily. Local ardor was the only event clearly related to the new developed product. It resolved spontaneously in 2-3 minutes and is more likely due to the excipients rather than to the PEG IFN alpha-2b molecule.

The process to obtain a stable and effective conjugation of IFN alpha-2 and branched PEG is complex. The details of the manufacturer are not disclosed, so it is likely that the procedures in a compared formulation are different. One of the most important features is the presence of different isomers of position, which have different biological activity since the interference between the surface receptor and the molecule IFN is different. This phenomenon can also affect the pharmacokinetic profile of PEG-IFN, since the covalent link remains until the elimination from the body. The difference in just one aminoacid between IFN alpha-2a and IFN alpha-2b should not determine differences in their biological properties but some affectations after the conjugation with PEG could not be discarded. In this trial both products were similar regarding their pharmacokinetic and pharmacodynamic profiles. In the case of newly developed PEG IFN alpha preparations, the comparable pharmacokinetics and pharmacodynamics data can be the basis for its further clinical development were efficacy and safety information can be gathered. Its introduction in the national health system will certainly have a significant impact on health care.

## Conclusion

The formulations have similar pharmacokinetic, pharmacodynamic and short-term safety profiles. Efficacy trials can be carried out to confirm clinical similarity.

## Competing interests

Authors IGG, CMVS, MCD, HNC, CHBB, IBR and PALS are employees of the Center for Biological Research, which is part of the Center for Genetic Engineering and Biotechnology (CIGB), Havana network, where IFN alpha-2b is produced and the new pegylated formulation was developed. JFB, RPM and FRCO are employees of CIGB itself. The rest of the authors have no competing interests at all. The study was financed by Heber Biotec, Havana, Cuba (products, reagents), and the Ministry of Public Health of Cuba (hospital facilities and general medical care of the volunteers as in-patients).

## Authors' contributions

IGG participated in the study design, coordination and performance, EIA determinations, analyses of results, and wrote the manuscript draft. CAGD, ADM and OPP took care of subject recruitment, management, clinical examinations, and follow-up. CMVS participated in the study design and statistical analysis. MCD, CHBB, JFB and IBR carried out laboratory determinations. HNC assisted as study monitor and results analyses. RPM and FRCO participated in study coordination and results analyses; PALS took part in the design, results analysis and manuscript writing. All authors read and approved the final manuscript.
